# Baseline serum neurofilament light chain levels differentiate aggressive from benign forms of relapsing–remitting multiple sclerosis: a 20-year follow-up cohort

**DOI:** 10.1007/s00415-023-12135-w

**Published:** 2023-12-12

**Authors:** Pablo Arroyo Pereiro, Albert Muñoz-Vendrell, Isabel León Moreno, Laura Bau, Elisabet Matas, Lucía Romero-Pinel, Antonio Martínez Yélamos, Sergio Martínez Yélamos, Pol Andrés-Benito

**Affiliations:** 1 Neurologic Diseases and Neurogenetics Group, Institute of Biomedical Research (IDIBELL), Avinguda de la Gran Via de L’Hospitalet, 199, L’Hospitalet de Llobregat, 08907 Barcelona, Spain; 2 Multiple Sclerosis Unit, Department of Neurology, Bellvitge University Hospital, L’Hospitalet de Llobregat, 08907 Barcelona, Spain; 3 Departament de Ciències Clíniques, Facultat de Medicina i Ciències de la Salut, Universitat de Barcelona (UB), Barcelona, Spain

**Keywords:** Multiple sclerosis, Biomarkers, Prognosis, sNfL, GFAP, CHI3L1

## Abstract

**Background and objectives:**

Serum biomarkers are emerging as useful prognostic tools for multiple sclerosis (MS); however, long-term studies are lacking. We aimed to evaluate the long-term prognostic value of the serum levels of neurofilament light chain (NfL), total tau, glial fibrillary acidic protein (GFAP), and chitinase 3-like-1 (CHI3L1) measured close to the time of MS onset.

**Methods:**

In this retrospective, exploratory, observational, case and controls study, patients with relapsing–remitting MS (RRMS) with available baseline serum samples and prospectively follow-up in our MS unit for a long time were selected based on their clinical evolution to form two groups: (1) a benign RRMS (bRRMS) group, defined as patients with an Expanded Disability Status Scale (EDSS) score of ≤ 3 at ≥ 10 years of follow-up; (2) an aggressive RRMS (aRRMS) group, defined as patients with an EDSS score of ≥ 6 at ≤ 15 years of follow-up. An age-matched healthy control (HC) group was selected. NfL, total tau, and GFAP serum levels were quantified using a single-molecule array (SIMOA), and CHI3L1 was quantified using ELISA.

**Results:**

Thirty-one patients with bRRMS, 19 with aRRMS, and 10 HC were included. The median follow-up time from sample collection was 17.74 years (interquartile range, 14.60–20.37). Bivariate and multivariate analyses revealed significantly higher NfL and GFAP levels in the aRRMS group than in the bRRMS group. A receiver operating characteristic curve analysis identified serum NfL level as the most efficient marker for distinguishing aRRMS from bRRMS.

**Discussion:**

This proof-of-concept study comparing benign and aggressive RRMS groups reinforces the potential role of baseline NfL serum levels as a promising long-term disability prognostic marker. In contrast, serum GFAP, total tau, and CHI3L1 levels demonstrated a lower or no ability to differentiate between the long-term outcomes of RRMS.

## Introduction

Multiple sclerosis (MS) is a chronic autoimmune disease characterized by multifocal inflammatory demyelination and neurodegeneration of the CNS, resulting in irreversible neurological damage and consequent accumulation of disability [1].

Disability progression shows great heterogeneity among patients, and it is believed that the early use of high-efficacy disease-modifying treatments (DMT) during the course of the disease could delay its irreversible accumulation in patients with high inflammatory activity [2–4]. Thus, identifying predictive markers of disability is becoming a priority as it will enable the selection of candidates for this treatment approach. Currently, there are few prognostic markers available, both clinical and radiological, with limited clinical utility [5–13], despite being combined in various scoring systems, wherein improving the predictive power involves incorporating variables from the early years of follow-up, which could result in missed opportunities for early treatment [14–16].

Accurate and specific biomarkers that targeting specific aspects of the pathological processes underlying MS are potential prognostic markers. The development of ultrasensitive digital immunoassays, such as the single molecule array (SIMOA), has enabled reliable measurement of CNS-relevant biomarkers in serum that were not previously detectable [17]. Neurofilament light chain (NfL), total tau protein, glial fibrillary acidic protein (GFAP), and chitinase 3-like-1 (CHI3L1) are promising prognostic biomarkers for MS. These biomarkers reflect neuronal damage occurring in acute and chronic lesions, astrocytic activation and astrogliosis, and oligodendrocytic activation at different stages of the disease, both in the inflammatory and neurodegenerative processes of MS [18–22]. Multiple studies have analyzed the relationship between disability progression and the baseline levels of NfL [23–26], GFAP [27–32], CHI3L1 [33–35], and total tau [36–41]. The results are inconsistent across different studies for NfL, GFAP, and total tau. Studies with follow-up periods exceeding 10 years are scarce.

The objective of this study was to investigate the predictive capacity of the serum biomarkers NfL, GFAP, CHI3L1, and total tau measured at the time of initial evaluation for long-term disability in patients with MS. Additionally, we aimed to compare these biomarkers to provide insights for future research.

## Methods

### Study design, participants, and clinical data collection

We designed an exploratory retrospective, longitudinal, observational, case and control study including patients diagnosed with remitting-relapsing MS (RRMS) according to the McDonald criteria of 2017 [42], who were prospectively followed up in the MS unit of a tertiary hospital with available serum samples at the time of initial patient evaluation. The STROBE guidelines for observational studies were used to conduct this study.

Two groups of patients were defined with distinctive inclusion criteria: a benign RRMS (bRRMS) group, defined as patients with an Expanded Disability Status Scale (EDSS) score of ≤ 3 at ≥ 10 years of follow-up, and an aggressive RRMS (aRRMS) group, defined as patients with an EDSS score of ≥ 6 at ≤ 15 years of follow-up. These inclusion criteria for each patient groups have been applied previously in other published works [43–46].

A third group of healthy controls (HC) were included, whose inclusion criteria were the absence of neurological diseases and the availability of a stored serum sample. They were selected by age matching to the patient groups.

The exclusion criteria were the presence of neurological comorbidities (peripheral or central nervous system) and relapse within the month before serum sample collection. In both patients and control groups, cases with samples not meeting the technical criteria (presence of particles, lipemia, or hemolyzed samples) were also excluded.

The European Database for Multiple Sclerosis software was used for the prospective collection of clinical data [47]. The variables collected were sex, date of birth, date of onset of MS symptoms, date of serum sampling, date of last relapse before serum sampling, date of last follow-up, total number of relapses during follow-up, EDSS at 10 years of onset for aRRMS, EDSS at 15 years of onset for bRRMS, EDSS at the end of follow-up for all patients, DMT received during follow-up, initiation date of each DMT, progression start date, baseline brain magnetic resonance imaging (MRI) date, presence of gadolinium-enhancing lesions on that MRI, and the presence of new lesions in T2 (in cases where possible, i.e., those with available previous MRI).

The following variables were calculated: age at MS onset, age at the time of serum sampling, total follow-up time, time from MS onset to serum sampling, time from previous relapse to serum sampling, time from baseline cerebral MRI to serum sampling, Multiple Sclerosis Severity Score (MSSS), and age-related Multiple Sclerosis Severity (ARMSS) scores. The DMT variable was analyzed as: “no DMT”; “moderate efficacy DMT” if none of the high efficacy DMT and any of the following was being used at any moment of the follow-up: interferon beta 1a, interferon beta 1b, peginterferon beta 1a, glatiramer acetate, teriflunomide, dimethyl fumarate; and “high efficacy DMT” if any of the following was being used at any moment of the follow-up: fingolimod, natalizumab, alemtuzumab, ocrelizumab, cladribine, rituximab, mitoxantrone.

### Serum collection, processing, and laboratory analysis

Patient and healthy control samples were provided by the Biobank HUB-ICO-IDIBELL, funded by the Instituto de Salud Carlos III (PT20/00171) and by Xarxa de Bancs de Tumors de Catalunya, sponsored by the Pla Director d’Oncologia de Catalunya (XBTC).

Serum samples were prospectively collected from patients undergoing blood extraction as part of the usual initial evaluation in the MS unit, using appropriate serum separation tubes. Once obtained, the serum was centrifuged at 3000 rpm for 15 min at room temperature, and the supernatant was collected, aliquoted in volumes of 500 μL, and stored at − 80 °C until analysis. HC samples were obtained from healthy volunteers without neurological disorders at the neurology service, following the same technical procedures.

Serum levels of total tau, GFAP, and NfL were measured in singlicate using NeuroPlex-4B kit (Quanterix, Billerica, MA, USA) and SIMOA technology (Quanterix, Billerica, MA, USA). CHI3L1 serum levels were analyzed using the MicroVue YKL-40 EIA kit from Quidel (Cat no. 8020, Quidel, CA, USA), following the manufacturer's instructions. All samples were analyzed in the same run, and all biomarker values fell within the detection range limits specified by the manufacturer.

In the case of serum neurofilament levels, the web application derived from the study by Benkert et al. was used to obtain scores of standard deviations (SD) relative to normal values of healthy controls. These scores were obtained by correcting only for age, as body mass index (BMI) at the time of sample collection was not available (in these cases, a BMI of 25 is assumed) [48].

### Study endpoints

The objectives of this study were to test whether serum NFL, GFAP, total tau, and CHI3L1 levels in the early course of MS were different between the bRRMS and aRRMS groups and to establish their ability to classify cases between the two groups.

### Statistical analysis

The distribution of data was assessed using the Shapiro–Wilk test. Data are described as mean ± standard deviation (SD) or as median and interquartile range (IQR) according to their distribution. Categorical variables were described using frequencies. Box plots were used to visualize the distribution of biomarkers across different groups. For the bivariate group comparison analysis, Fisher’s exact test, Mann–Whitney *U* test, Kruskal–Wallis test, and Poisson or negative binomial regression were applied as appropriate. If the Kruskal–Wallis test was rejected, Dunn’s pairwise multiple comparison test was applied. For multivariate analysis using the MS patient groups, a binary logistic regression model was performed, including all available clinical variables that could act as confounders as independent variables. Diagnostic tests were performed for the regression model to avoid nonlinearity between the predictor variables and the odds logit and collinearity between the predictor variables. For the binomial logistic regression analysis, a logarithmic transformation was applied to the biomarker data to obtain a normal distribution and linearity with the odds ratio logit, as it has been shown that they follow a lognormal distribution. This logarithmic transformation was done according to the equation “ln (*X* − *k*)” where “*X*” is the continuous independent variable and “*k*” was calculated to achieve a skewness of 0. To assess the biomarker accuracy in classifying patients into benign or aggressive forms, receiver operating characteristic (ROC) curves and the derived area under the curve (AUC) were calculated. Outliers were not excluded from the analysis. The best cutoff value, sensitivity, and specificity were estimated based on the Youden index [49]. And ROC curve standard error calculations, and comparisons were performed using DeLong’s method [50, 51]. All tests were conducted with 95% confidence intervals (CI) and a significance level of 5%. Bonferroni adjustment was used for multiple comparisons in the Dunn test and ROC curve comparison test. In order to maintain the same *p* value threshold across all test and improve readability, this was done multiplying the resulting *p* value from each comparison within a multiple comparison by “*m*”, where “*m*” is the number of comparisons performed. Statistical analysis and visualization were performed using Stata 18 software (StataCorp LLC, Texas, USA) and GraphPad Prism version 9.5.0 (La Jolla, CA, USA).

### Standard protocol approvals, registrations, and patient consents

This study was approved by the Ethics Committee (CEIC) of the Hospital Universitari de Bellvitge (reference number PR257/23). Patient information confidentiality was addressed in accordance with Spanish regulations. All participants provided written informed consent before sample storage in the IDBELL-ICO-HUB Biobank, following the guidelines of Spanish legislation on this matter (Real Decreto 1716/2011) and the approval of the CEIC of Bellvitge University Hospital.

### Data availability statement

Anonymized data not published within this article will be made available by request from any qualified investigator.

## Results

### Study population and groups’ characteristics

Fifty patients with RRMS (31 with bRRMS and 19 with aRRMS) and 10 age-matched HC were included in this study. The baseline demographic characteristics were similar between the two groups of RRMS patients and healthy controls, including age at the time of serum sampling (median of 36 completed years in the bRRMM vs. 43 years in the aRRMS vs. 40.5 years in the HC groups, *p* = 0.25) and sex ratio (74.2% were female in the bRRMS group vs. 47.4% in aRRMS vs. 50% in HC, *p* = 0.135).

The clinical characteristics and comparison tests between the two groups of patients with RRMS are summarized in Table 1. Globally, 64% of patients were female, and the mean age of MS onset was 36.6 ± 9 years old. They were followed-up for a median of 17.74 years (IQR, 14.60–20.37) out of a total median disease duration of 20.37 years (IQR, 17.97–23.51). Although no statistically significant differences were found in the age at MS onset or sex ratios, a lower proportion of females was observed in the aRRMS group (74.2% in the bRRMS vs. 47.4% in the aRRMS group, *p* = 0.073). The total follow-up time (median of 18.29 years in bRRMS vs 15.01 in aRRMS, *p* = 0.03) and disease duration (median of 20.86 years in bRRMS vs 15.89 in aRRMS, *p* = 0.03) were significantly longer in the bRRMS group. The median EDSS score at the time of serum collection was 1.5 (1 for bRRMS vs. 3 for aRRMS; *p* < 0.001). The median EDSS at the last follow-up was 1.5 (IQR, 1.5–2) for the bRRMS group and 7.5 (IQR, 7–8) for the aRRMS group, and both MSSS and ARMSS score were significantly higher in aRRMS group (*p* < 0.001). No differences were found between the two groups in terms of age at the time of sample collection, time from MS onset to sample collection, or time from prior relapse to sample collection.

**Table 1:** Clinical characteristics and group comparisons

	All patients	Benign MS	Aggressive MS	*p* value
Number of patients	50	31	19	
Age at MS onset, years (mean ± SD)	36.6 ± 9	35.7 ± 7.2	38 ± 11.4	0.58^A^
Female, *n* (%)	32 (64%)	23 (74.2%)	9 (47.4%)	0.073^B^
Disease duration (from onset to last follow-up), years [median (IQR)]	20.37 (17.97–23.51)	20.86 (19.05–23.67)	15.89 (13.64–22.25)	0.03*^A^
Follow-up time from serum sampling, years [median (IQR)]	17.74 (14.60–20.37)	18.29 (16.61–20.50)	15.01 (9.58–20.12)	0.03*^A^
ARR [median (IQR)]	0.26(0.15–0.46)	0.17 (0.15–0.35)	0.49 (0.2–0.73)	0.002*^C^
EDSS at last follow-up [median (IQR)]	2 (1.5–7.5)	1.5 (1.5–2)	7.5 (7–8)	< 0.001*^A^
MSSS at last follow-up (mean ± SD)	3.73 ± 3.93	0.72 ± 0.3	8.65 ± 0.87	< 0.001*^A^
ARMSS score at last follow-up (mean ± SD)	4.19 ± 3.81	1.28 ± 0.63	8.95 ± 0.69	< 0.001*^A^
DMT during follow-up, *n* (%)				0.006*^B^
No DMT Moderate efficacy DMT High efficacy DMT	8 (16%) 32 (64%) 10 (20%)	7 (22.58%) 22 (70.97%) 2 (6.45%)	1 (5.26%) 10 (52.63%) 8 (42.11%)	
SPMS during follow-up, *n* (%)	17 (34%)	0 (0%)	17 (89.47%)	< 0.001*^B^
Age at serum sampling, years (mean ± SD)	39.55 ± 9.52	38.67 ± 8.46	40.97 ± 11.12	0.48^A^
EDSS at serum sampling [median (IQR)]	1.5 (1–2.5)	1 (0–1.5)	3 (2–3.5)	< 0.001*^A^
Time from MS onset to serum sampling, months [median (IQR)]	21.08 (8.18–48.3)	19.68 (5.88–54.57)	27.86 (9.2–48.3)	0.69^A^
Time from previous relapse to serum sampling, months [median (IQR)]	6.46 (3.71–21.88)	5.85 (3.75–28.25)	7.13 (3–19.02)	0.49^A^

*MS* multiple sclerosis, *SD* standard deviation, *IQR* interquartile range, *ARR* annualized relapse rate, *EDSS* Expanded Disability Status Scale, *MSSS* multiple sclerosis severity score, *ARMSS* age-related multiple sclerosis severity score, *DMT* disease-modifying treatment, *SPMS* secondary progressive multiple sclerosis

*Statistically significant (*p* < 0.05)

^A^Mann Whitney *U* test

^B^Fisher’s exact test

^C^Negative binomial regression

Baseline cerebral MRI data were available for 48 out of 50 patients (1 missing in each group). The median time between the acquisition of these baseline MRIs and the serum sample collection was 3.35 months (IQR 0.54–23.3), with no statistically significant differences between the two patient groups (median 4.40 months in bRRMS vs. 1.49 in aRRMS, *p* = 0.073 by Mann–Whitney test). In 8 out of these 48 cases, a previous brain MRI allowed estimating the appearance of new T2 lesions (4 in the bRRMS group and 4 in the aRRMS group); in the other 40 cases, the presence of radiological activity could only be assessed by gadolinium enhancement as it was the first MRI performed. Radiological activity was observed in a total of five baseline MRIs: two in the bRRMS group (two due to gadolinium enhancement and another due to new T2 lesions) and three in the aRRMS group (all due to gadolinium enhancement), with no statistically significant differences between groups (*p* = 0.349 by Fisher’s test).

### Serum biomarkers in aggressive and benign groups

**Fig. 1:**
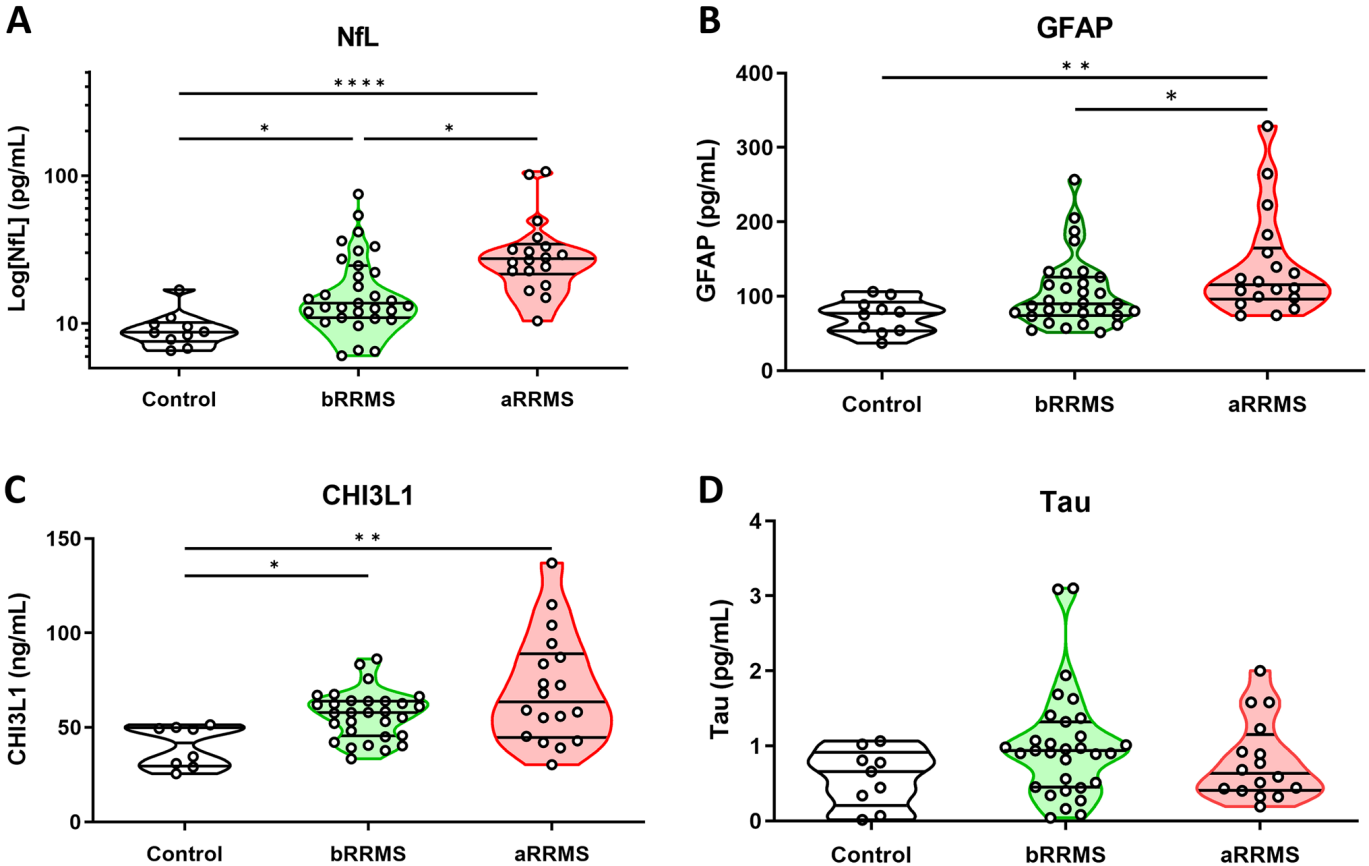
Distribution boxplots of NfL (**A**), GFAP (**B**), CHI3L1 (**C**), and total Tau (**D**) levels in the serum of benign and aggressive forms of RRMS patients and control cases using single molecule array (SIMOA) and ELISA technologies. A case scatter plot is represented by dots and outliers by triangles. A logarithmic scale for NFL levels was used in **A** to improve data visualization. Statistical signification (corrected for multiple comparisons in Dunn test) among groups were set at *p < 0.05, **p < 0.01, ***p < 0.001 and ****p < 0.0001

The serum biomarker levels of the three groups and the results of the bivariate contrast tests are summarized in Table 2. Figure 1 shows boxplots of serum biomarker levels. Statistically significant differences in NfL, GFAP, and CHI3L1 serum levels between at least two groups were found in bivariate analysis (*p* < 0.001, *p* = 0.002, and *p* = 0.003, respectively). For serum NfL levels, Dunn’s test confirmed that all groups were significantly different (HC vs. bRRMS, *p* = 0.0079; HC vs. aRRMS, *p* < 0.001; and bRRMS vs. aRRMS, *p* = 0.0066). In the case of GFAP serum levels, the aRRMS group levels were significantly different from those in the HC and bRRMS groups (*p* < 0.001 and *p* = 0.047, respectively); however, there were no significant differences between the HC and bRRMS groups (*p* = 0.072). CHI3L1 levels were significantly different in the HC group from those in both bRRMS and aRRMS patients (*p* = 0.013 and *p* = 0.001, respectively); however, there were no differences between the bRRMS and aRRMS patients (*p* = 0.31).

**Table 2:** Biomarkers and groups bivariate tests

	All participants	Healthy controls	Benign RRMS	Aggressive RRMS	H	*p* value
NfL, pg/ml [median (IQR)] Sample size	15.34 (10.59–27.97) 59	8.74 (7.83–9.89) 10	13.73 (10.96–24.66) 31	27.44 (22.71–33.17) 18	22.69	< 0.001*
GFAP, pg/ml [median (IQR)] Sample size	94.52 (75.21–125.86) 59	77.24 (54.26–88.59) 10	89.94 (74.22–125.86) 31	115.44 (98.18–158.81) 18	12.20	0.002*
CHI3L1, pg/ml [median (IQR)] Sample size	56.76 (44.02–66.7) 56	41.80 (29.98–49.76) 8	57.95 (45.66–63.9) 30	63.62 (45.33–87.24) 18	11.27	0.003*
Tau, pg/ml [median (IQR)] Sample size	0.86 (0.44–1.07) 56	0.66 (0.34–0.81) 9	0.94 (0.45–1.32) 31	0.64 (0.42–1.08) 16	3.64	0.16

*MS* multiple sclerosis, *H* Kruskal–Wallis analysis-of-variance test, *NfL* neurofilament light chain serum levels, *GFAP* glial fibrillary acidic protein serum levels, *CHI3L1* CHI3L1 protein serum levels, *Tau* total serum tau levels, *IQR* interquartile range

*Statistically significant (*p* < 0.05)

In the case of neurofilaments, the analysis was repeated using SD scores relative to normal values from a large cohort of healthy subjects [48]. The control, bRRMS and aRRMS groups had a median score in standard deviation of 0.9 (IQR 0.18–1.23), 2.05 (IQR 1.68–2.85), and 2.68 (IQR 2.46–3.09), respectively. Similar to absolute values, the Kruskal–Wallis test (*p* < 0.001) and pairwise comparisons using the Dunn test confirmed that SD of NfL levels were different among all groups (control vs bRRMS *p* = 0.004, control vs aRRMS *p* < 0.001, bRRMS vs aRRMS *p* = 0.01).

### Benign and aggressive multiple sclerosis classification accuracy of serum biomarkers

**Fig. 2:**
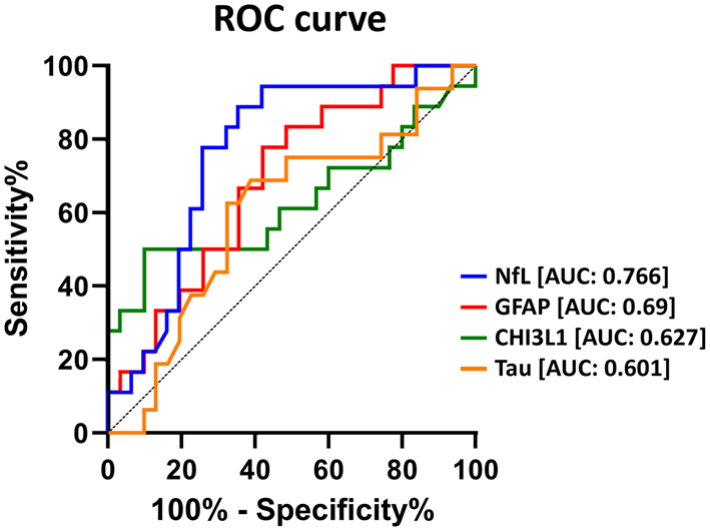
Receiver operating characteristic (ROC) curves of NfL, GFAP, CHI3L1 and total Tau serum quantification in the differential diagnosis of benign RRMS and aggressive RRMS. AUC values, corresponding to the area under ROC curves

The ROC curve analysis results assessing the bRRMS vs aRRMS classification power are summarized in Table 3 and Fig. 2. Of note, the only biomarkers with an AUC greater than 0.5 that were statistically significant were NfL and GFAP. When performing multiple comparisons among the biomarker curves, the AUC of NfL and GFAP were significantly higher than those of total tau (*p* = 0.020 and 0.006, respectively). No significant differences were found between the AUCs of NfL and GFAP (*p* = 1) or between those of CHI3L1 and total tau (*p* = 0.1). When conducting the ROC curve analysis to assess the discriminative capacity between bRRMS and aRRMS groups using SD scores for NfL serum levels, the AUC was very similar to that obtained using absolute values, with no statistically significant differences between the two (AUC 0.753 with SD vs 0.766 using absolute values, *p* = 0.63). The best NfL SD score cut-off value based on Youden index was 2.26.

**Table 3:** ROC curve analysis for bMMRS and aMMRS classification accuracy of basal serum biomarkers

	AUC (95% CI)	Optimal cut-off^a^ (pg/ml)	Sensitivity (%)	Specificity (%)
NfL	0.766 (0.628–0.904)	15.63	88.89	64.52
GFAP	0.69 (0.539–0.841)	94.52	77.78	58.06
CHI3L1	0.627 (0.442–0.812)	67.61	50	90
Tau	0.601 (0.425–0.777)	0.82	62.5	67.7

*AUC* area under the curve, *CI* confidence interval, *NfL* neurofilament light chain, *GFAP* glial fibrillary acidic protein, *CHI3L1* chitinase 3-like-1

^a^By Youden Index

In the multivariate binary logistic regression analysis, age at the time of serum sampling, EDSS score at the time of serum sampling, and time from previous relapse to serum sampling were included as covariables. This association remained statistically significant after multivariate adjustment for NfL. The overall NfL model showed a good fit to the data (LL(M) = − 16.59) and statistical significance (*χ*^2^(*df* = 4) = 31.26, *p* < 0.001), and the estimated odds ratio for predictor variable NfL was 3.67 (95% confidence interval 1.14–11.87; *X*^2^ Wald = 4.71, *p* = 0.03), indicating that NfL was an independent predictor of the outcome. The remaining biomarkers (GFAP, CHI3L1, and total tau) did not show a significant association in multivariate binary logistic regression analysis.

## Discussion

This proof-of-concept study reported an association between serum NfL and GFAP levels measured early in the course of MS and subsequent EDSS progression, showing acceptable predictive power to differentiate two groups of benign and aggressive forms of RRMS in patients with a disease duration of 20 years and an average follow-up period of approximately 18 years.

Baseline serum NfL levels have been associated with worsening of EDSS in the first year, number of relapses, and progression of brain atrophy, as measured by cerebral magnetic resonance imaging (MRI) [52–55] in short-term prognosis studies. Regarding long-term prognosis, both baseline and longitudinal measures of NfL levels have been linked to greater cerebral MRI-based brain atrophy [25, 53, 56–60]; however, their association with disability progression is inconsistent [23]. In fact, studies with longer clinical follow-up periods found no relationship between baseline NfL levels and disability progression [24, 25]. However, a recent study with a median follow-up of approximately 7 years found an association between baseline NfL levels and a higher risk of confirmed disability progression with an EDSS ≥ 3 during the follow-up period [26]. Additionally, there is a previous study for NfL with a similar design as that of ours, including patients with RRMS, that also demonstrates that baseline sNfL is associated with long term (18.9 years) clinical disease progression and may be a sensitive marker of subsequent poor clinical outcomes [61]. It is worth noting that this study includes clinically isolated syndrome (CIS) and primary progressive MS (PPMS) in addition to RRMS in its cohort, which could have potentially influenced a positive outcome.

Regarding the basal serum GFAP levels, the first study in 2019 failed to demonstrate the prognostic value of GFAP in predicting outcomes [27]. Subsequently, two studies, with median follow-up periods of 3.1 and 4.4 years and smaller cohorts (94 and 115 patients, respectively), found no relationship between GFAP and disability measured using different scales [28, 29]. Two other studies with larger cohorts (257 and 355 patients) and longer follow-up periods (approximately 7 years) found a relationship between GFAP and disease progression [30, 31]. One study demonstrated that GFAP, measured at baseline and longitudinally in subsequent measurements, is a good predictor of disease progression independent of relapse activity (PIRA) [31]. However, a recent study analyzing the prognostic potential of GFAP and PIRA found little or no correlation [32]. Finally, a recent study demonstrated that baseline serum levels of GFAP, either alone or in combination with baseline NfL serum levels, as well as its levels measured after 1 year of follow-up, improved the performance of predictive models that included all known clinical and radiological prognostic variables for EDSS outcomes at 10 years [62].

Our study supports the idea that baseline serum NfL and GFAP levels are predictors of long-term disability progression. The results of the ROC curves also suggest that NfL has a better capacity than GFAP to differentiate between the bRRMS and aRRMS groups and, therefore, has a better prognostic ability, although the AUC comparison test failed to demonstrate a statistically significant difference between both, possibly due to a lack of statistical power. The serum NfL level was probably the most promising biomarker in this study a priori, given that it is a neuronal cytoskeletal component involved in axonal transport and is considered a good surrogate marker of neuronal axonal damage and, therefore, of the lesion burden at the time of its determination, even though it is non-specifically elevated in multiple pathologies [18]. Although it is considered a marker of neuronal damage due to active lesions, a recent study demonstrated that its levels are capable of predicting disability progression independent of relapses in patients undergoing treatment with ocrelizumab [63]. GFAP is the main intermediate filament of human astrocytes and is considered a marker of astrocytic injury and astrogliosis [64]. The idea that GFAP could be a good marker of pure progression has been considered because of its high levels in progressive forms, both in PPMS and secondary progressive MS (SPMS) without recent inflammatory activity [34, 65]. Notably, due to its design, this study does not help clarify whether basal GFAP is related to disability through PIRA, for which contradictory results exist [31, 32].This may explain why previous studies with a shorter follow-up period did not find a relationship between baseline GFAP and prognosis in RRMS patients, unlike those with a longer follow-up period.

In multivariate analysis, NfL association remained statistically significant after accounting for confounding effects such as time between the last relapse and serum collection, and known clinical prognostic markers such as age and EDSS at the moment of serum sampling. An interesting characteristic of our cohort is that being composed of patients with long-term follow-up, serum NfL and GFAP levels were minimally influenced by the early use of high-efficacy treatments as this strategy had not yet been implemented. In fact, at the time of sample collection and initiation of follow-up, only 11 out of 50 patients received moderately effective DMT, and none received highly effective DMT. This is a factor should be considered when interpreting our results as some studies have demonstrated the influence of DMT on serum NfL and GFAP levels, especially in patients on high efficacy DMT [48, 66, 67]. Surprisingly, in our sample, benign forms had a higher proportion of patients under treatment at the time of sample extraction: 10 in bRRMS vs. 1 in aRRMS. From those, 8 were on interferon beta-1a 22ug 3 times per week, 1 on interferon beta-1a 30ug once a week and 1 on glatiramer acetate in the bRRMS group, and 1 was on cyclophosphamide in the aRRMS group. However, the levels of NfL and GFAP in the treated bRRMS group and the untreated bRRMS group were not significantly different (median of 12.44 vs 15.34, *p* = 0.15). Moreover, when conducting a sensitivity analysis comparing bRRMS and aRRMS by excluding those 11 patients under treatment, the results remained consistent in the bivariate test for both NfL (*p* = 0.029) and GFAP (*p* = 0.026), and the ROC curve analysis (AUC = 0.71 for both NfL and GFAP). Another noteworthy characteristic of this cohort is that only two patients from the bRRMS group and three from the aRRMS group exhibited radiological activity on the baseline brain MRI. Therefore, it is unlikely that this activity significantly influenced the mean baseline levels of NfL and GFAP in the groups, thus minimizing bias in the results.

Concerning the adjustment of serum NfL levels based on age, as suggested by several studies, for this specific use, the results of the comparative analysis using absolute values and standard deviation scores did not show significant differences between the two, in line with what was suggested by a meta-analysis that supported the notion that this effect is diluted under pathological conditions such as MS [68]. Additionally, age at the time of sample collection was not significantly different between the study groups, and this variable did not influence the results of the multivariable models (data not shown). Regarding the possible influence of previous relapses on the sample extraction over NfL levels [48], the median time between both events was 6.46 months (IQR, 3.71–21.88), with no statistically significant differences between the groups. Furthermore, inclusion in the multivariate model did not significantly affect the results.

In the GFAP model, the introduction of EDSS at the time of extraction rendered the serum GFAP levels insignificant. In our experience, GFAP has not demonstrated the ability to discriminate between patients with benign and aggressive forms of MS after a follow-up period of approximately 18 years. However, in other studies with shorter follow-up periods, GFAP appeared to have some predictive capacity [62].

CHI3L1 and tau levels were not significantly different between the benign and aggressive groups, and their classification power was not deemed acceptable. Therefore, unlike some previous studies, our study failed to demonstrate a relationship between baseline serum CHI3L1 and total tau levels.

There are few studies on the predictive power of basal levels of CHI3L1 for disability prognosis, all of which show positive results but have limited clinical follow-up and, most importantly, CSF samples rather than serum samples [33–35]. This fact should be taken into account, as there is evidence that systemic levels of CHI3L1 can increase in a variety of non-neurological pathologies [69].The study with the longer follow-up (median, 11.7 years) in a cohort of 301 patients with RRMS and CIS showed that elevated levels of CHI3L1 at disease onset independently predicted a shorter time to reach irreversible EDSS score of 3 and 6 [34]. An association was also demonstrated between baseline CHI3L1 levels and EDSS at 1 year in a small cohort of patients with PPMS [35]. It is worth mentioning that although progressive forms of MS, especially PPMS, have been associated with elevated levels of CHI3L1 in both CSF and serum [70, 71], this does not necessarily imply that baseline serum levels can be predictive.

Studies on total tau and prognosis in MS, are scarce and have been conducted with short follow-up periods, and the results are inconclusive. Furthermore, in most of these studies, total tau levels were determined in the CSF using ELISA. One study related baseline CSF total tau levels to the MSSS and ARMSS with a 2-year follow-up [36], whereas another study linked these levels to the final EDSS score with a 3-year follow-up [37]. However, several studies have failed to demonstrate this relationship [38–41]. There are no studies in serum for total tau, and although in this case, the determination was performed using SIMOA, it is noteworthy that the levels are over 100 times higher in CSF [36, 37] than in serum, resulting in a median total tau level in serum of 0.86 pg/mL in the 56 participants of our study, with little variation between groups. Similar to other neurological disorders where total tau in CSF has demonstrated its utility, these results were not replicated in serum [72].

As an exploratory observational study, the rationale behind exclusively including the extremes of the population spectrum regarding disability progression was to maximize sensitivity and, therefore, cost-effectiveness of the study. Regarding the selection criteria for patient groups, the definition criterion for bRRMS is more widely accepted despite multiple definitions and a lack of consensus for aRRMS [43–45]. In our study, we chose a previously used criterion that was effective and allowed us to obtain a sufficient sample size [46]. However, this design also implies a series of limitations. The study is not suitable for providing odds ratios (OR) because it does not include the entire cohort of patients; therefore, these are pseudo-odds ratios. Nevertheless, this study provides valuable information regarding the potential of biomarkers, especially their comparative analysis, to guide future studies.

For the same reason, this observed prognostic capacity is undoubtedly overestimated, as the inclusion of an unselected sample will yield more overlapping levels of NfL and GFAP. Consequently, our study did not allow us to assert the usefulness of these biomarkers in clinical practice. In addition to the limitations inherent to observational studies and their designs, two other limitations should be highlighted. The first was the absence of follow-up radiological data, which, although not necessary for the intended purpose of evaluating and comparing the predictive power of the baseline levels of the biomarkers, would have been of great interest. The second stems from its sample size, although considered sufficient to ensure reliable observational results, which restricted the number of covariates that could be included in the multivariable models. The results of our multivariate analysis for NfL support the idea that it is a robust prognostic marker, however, further research including other previously described prognostic markers, both clinical and radiological, and a complete sample of patients will be necessary to confirm their utility and that it is an independent prognostic marker.

In conclusion, this study demonstrated that serum levels of GFAP and especially NfL, measured at the time of initial evaluation have potential to predict long-term disability in patients with MS. However, the roles of total tau and CHI3L1 remain uncertain. Therefore, serum GFAP and NfL levels should be considered candidates for inclusion in scoring systems along with other variables aimed at predicting long-term disability.

## References

[1] Reich DS, Lucchinetti CF, Calabresi PA (2018). Multiple sclerosis. N Engl J Med.

[2] Weideman AM, Tapia-Maltos MA, Johnson K, Greenwood M, Bielekova B (2017). Meta-analysis of the age-dependent efficacy of multiple sclerosis treatments. Front Neurol.

[3] Ontaneda D, Tallantyre E, Kalincik T, Planchon SM, Evangelou N (2019). Early highly effective versus escalation treatment approaches in relapsing multiple sclerosis. Lancet Neurol.

[4] Harding K (2019). Clinical outcomes of escalation vs early intensive disease-modifying therapy in patients with multiple sclerosis. JAMA Neurol.

[5] Paul A, Comabella M, Gandhi R (2019). Biomarkers in multiple sclerosis. Cold Spring Harb Perspect Med.

[6] Tintore M (2015). Defining high, medium and low impact prognostic factors for developing multiple sclerosis. Brain.

[7] D’Amico E, Patti F, Leone C, Lo Fermo S, Zappia M (2016). Negative prognostic impact of MRI spinal lesions in the early stages of relapsing–remitting multiple sclerosis. Mult Scler J Exp Transl Clin.

[8] Fisniku LK (2008). Disability and T 2 MRI lesions: a 20-year follow-up of patients with relapse onset of multiple sclerosis. Brain.

[9] Swanton JK (2009). Early MRI in optic neuritis: the risk for disability. Neurology.

[10] Minneboo A, Barkhof F, Polman CH, Uitdehaag BMJ, Knol DL, Castelijns JA (2004). Infratentorial lesions predict long-term disability in patients with initial findings suggestive of multiple sclerosis. Arch Neurol.

[11] Malpas CB (2020). Early clinical markers of aggressive multiple sclerosis. Brain.

[12] Jokubaitis VG (2016). Predictors of long-term disability accrual in relapse-onset multiple sclerosis. Ann Neurol.

[13] Dekker I (2020). Infratentorial and spinal cord lesions: cumulative predictors of long-term disability?. Mult Scler J.

[14] Pisani AI, Scalfari A, Crescenzo F, Romualdi C, Calabrese M (2021). A novel prognostic score to assess the risk of progression in relapsing−remitting multiple sclerosis patients. Eur J Neurol.

[15] Gasperini C (2021). Scoring the 10-year risk of ambulatory disability in multiple sclerosis: the RoAD score. Eur J Neurol.

[16] Sormani MP (2013). Scoring treatment response in patients with relapsing multiple sclerosis. Mult Scler J.

[17] Comabella M, Sastre-Garriga J, Montalban X (2016). Precision medicine in multiple sclerosis: biomarkers for diagnosis, prognosis, and treatment response. Curr Opin Neurol.

[18] Gafson AR (2020). Neurofilaments: neurobiological foundations for biomarker applications. Brain.

[19] Abdelhak A (2022). Blood GFAP as an emerging biomarker in brain and spinal cord disorders. Nat Rev Neurol.

[20] Bonneh-Barkay D, Wang G, Starkey A, Hamilton RL, Wiley CA (2010). In vivo CHI3L1 (YKL-40) expression in astrocytes in acute and chronic neurological diseases. J Neuroinflamm.

[21] Starossom SC (2019). Chi3l3 induces oligodendrogenesis in an experimental model of autoimmune neuroinflammation. Nat Commun.

[22] Hampel H, Blennow K, Shaw LM, Hoessler YC, Zetterberg H, Trojanowski JQ (2010). Total and phosphorylated tau protein as biological markers of Alzheimer’s disease. Exp Gerontol.

[23] Manouchehrinia A (2020). Plasma neurofilament light levels are associated with risk of disability in multiple sclerosis. Neurology.

[24] Aloizou AM (2022). Baseline neurofilament levels in cerebrospinal fluid do not correlate with long-term prognosis in multiple sclerosis. Mult Scler Relat Disord.

[25] Cantó E (2019). Association between serum neurofilament light chain levels and long-term disease course among patients with multiple sclerosis followed up for 12 years. JAMA Neurol.

[26] Monreal E (2023). Association of serum neurofilament light chain levels at disease onset with disability worsening in patients with a first demyelinating multiple sclerosis event not treated with high-efficacy drugs. JAMA Neurol.

[27] Watanabe M (2019). Serum GFAP and neurofilament light as biomarkers of disease activity and disability in NMOSD. Neurology.

[28] Barro C (2022). Serum NfL but not GFAP predicts cognitive decline in active progressive multiple sclerosis patients. Mult Scler J.

[29] Pauwels A (2022). Plasma glial fibrillary acidic protein and neurofilament light chain in relation to disability worsening in multiple sclerosis. Mult Scler.

[30] Barro C (2022). Serum GFAP and NfL levels differentiate subsequent progression and disease activity in patients with progressive multiple sclerosis. Neurol Neuroimmunol Neuroinflamm.

[31] Meier S (2023). Serum glial fibrillary acidic protein compared with neurofilament light chain as a biomarker for disease progression in multiple sclerosis. JAMA Neurol.

[32] Jiang X (2023). Glial fibrillary acidic protein and multiple sclerosis progression independent of acute inflammation. Mult Scler J.

[33] Lucchini M (2023). CSF CXCL13 and chitinase 3-like-1 levels predict disease course in relapsing multiple sclerosis. Mol Neurobiol.

[34] Martínez MAM (2015). Glial and neuronal markers in cerebrospinal fluid predict progression in multiple sclerosis. Mult Scler J.

[35] Pérez-Miralles F (2020). CSF chitinase 3-like-1 association with disability of primary progressive MS. Neurol Neuroimmunol Neuroinflamm.

[36] Virgilio E (2021). Cerebrospinal tau levels as a predictor of early disability in multiple sclerosis. Mult Scler Relat Disord.

[37] Martínez-Yélamos A, Saiz A, Bas J, Hernandez JJ, Graus F, Arbizu T (2004). Tau protein in cerebrospinal fluid: a possible marker of poor outcome in patients with early relapsing-remitting multiple sclerosis. Neurosci Lett.

[38] Brettschneider J (2005). Tau protein level in cerebrospinal fluid is increased in patients with early multiple sclerosis. Mult Scler.

[39] Guimarães J, Cardoso MJ, Sá MJ (2006). Tau protein seems not to be a useful routine clinical marker of axonal damage in multiple sclerosis. Mult Scler.

[40] HeinNéeMaier K (2008). Biological markers for axonal degeneration in CSF and blood of patients with the first event indicative for multiple sclerosis. Neurosci Lett.

[41] Terzi M, Birinci A, Çetinkaya E, Onar MK (2007). Cerebrospinal fluid total tau protein levels in patients with multiple sclerosis. Acta Neurol Scand.

[42] Thompson AJ (2018). Diagnosis of multiple sclerosis: 2017 revisions of the McDonald criteria. Lancet Neurol.

[43] Iacobaeus E (2020). Aggressive multiple sclerosis (1): towards a definition of the phenotype. Mult Scler J.

[44] Glad SB, Aarseth JH, Nyland H, Riise T, Myhr KM (2010). Benign multiple sclerosis: a need for a consensus. Acta Neurol Scand.

[45] Arroyo-Pereiro P (2023). Kappa free light chains index in multiple sclerosis very long-term prognosis. Front Immunol.

[46] Tintore M (2020). The long-term outcomes of CIS patients in the Barcelona inception cohort: looking back to recognize aggressive MS. Mult Scler J.

[47] Confavreux C, Compston DAS, Hommes OR, McDonald WI, Thompson AJ (1992). EDMUS, a European database for multiple sclerosis. J Neurol Neurosurg Psychiatry.

[48] Benkert P (2022). Serum neurofilament light chain for individual prognostication of disease activity in people with multiple sclerosis: a retrospective modelling and validation study. Lancet Neurol.

[49] Hughes G (2015). Youden’s index and the weight of evidence revisited. Methods Inf Med.

[50] Zhang DD, Zhou XH, Freeman DH, Freeman JL (2002). A non-parametric method for the comparison of partial areas under ROC curves and its application to large health care data sets. Stat Med.

[51] Bantis LE (2023). Statistical assessment of the prognostic and the predictive value of biomarkers—a biomarker assessment framework with applications to traumatic brain injury biomarker studies. Res Methods Med Health Sci.

[52] Disanto G (2017). Serum neurofilament light: a biomarker of neuronal damage in multiple sclerosis. Ann Neurol.

[53] Barro C (2018). Serum neurofilament as a predictor of disease worsening and brain and spinal cord atrophy in multiple sclerosis. Brain.

[54] Calabresi PA (2021). Temporal profile of serum neurofilament light in multiple sclerosis: implications for patient monitoring. Mult Scler.

[55] Thebault S (2022). High or increasing serum NfL is predictive of impending multiple sclerosis relapses. Mult Scler Relat Disord.

[56] Kuhle J (2017). Serum neurofilament is associated with progression of brain atrophy and disability in early MS from Neurologic Clinic and Policlinic. Neurology.

[57] Chitnis T (2018). Neurofilament light chain serum levels correlate with 10-year MRI outcomes in multiple sclerosis. Ann Clin Transl Neurol.

[58] Jakimovski D (2019). Serum neurofilament light chain levels associations with gray matter pathology: a 5-year longitudinal study. Ann Clin Transl Neurol.

[59] Srpova B (2021). Serum neurofilament light chain reflects inflammation-driven neurodegeneration and predicts delayed brain volume loss in early stage of multiple sclerosis. Mult Scler J.

[60] Buchmann A (2023). High serum neurofilament light chain levels correlate with brain atrophy and physical disability in multiple sclerosis. Eur J Neurol.

[61] Thebault S, Abdoli M, Fereshtehnejad SM, Tessier D, Tabard-Cossa V, Freedman MS (2020). Serum neurofilament light chain predicts long term clinical outcomes in multiple sclerosis. Sci Rep.

[62] Bose G (2023). Early neurofilament light and glial fibrillary acidic protein levels improve predictive models of multiple sclerosis outcomes. Mult Scler Relat Disord.

[63] Bar-Or A (2023). Blood neurofilament light levels predict non-relapsing progression following anti-CD20 therapy in relapsing and primary progressive multiple sclerosis: findings from the ocrelizumab randomised, double-blind phase 3 clinical trials. EBioMedicine.

[64] Eng LF, Ghirnikar RS, Lee YL (2000). Glial fibrillary acidic protein: GFAP-thirty-one years (1969–2000). Neurochem Res.

[65] Norgren N, Sundström P, Svenningsson A, Rosengren L, Stigbrand T, Gunnarsson M (2004). Neurofilament and glial fibrillary acidic protein in multiple sclerosis. Neurology.

[66] Delcoigne B (2020). Blood neurofilament light levels segregate treatment effects in multiple sclerosis. Neurology.

[67] Högel H (2020). Serum glial fibrillary acidic protein correlates with multiple sclerosis disease severity. Mult Scler.

[68] Bridel C (2019). Diagnostic value of cerebrospinal fluid neurofilament light protein in neurology: a systematic review and meta-analysis. JAMA Neurol.

[69] Pinteac R, Montalban X, Comabella M (2020). Chitinases and chitinase-like proteins as biomarkers in neurologic disorders. Neurol Neuroimmunol Neuroinflamm.

[70] Mañé-Martínez MA (2016). Glial and neuronal markers in cerebrospinal fluid in different types of multiple sclerosis. J Neuroimmunol.

[71] Cantó E (2011). Chitinase 3-like 1 plasma levels are increased in patients with progressive forms of multiple sclerosis. Mult Scler.

[72] Illán-Gala I (2021). Plasma tau and neurofilament light in frontotemporal lobar degeneration and Alzheimer disease. Neurology.

